# S-Nitrosylation of α1-Antitrypsin Triggers Macrophages Toward Inflammatory Phenotype and Enhances Intra-Cellular Bacteria Elimination

**DOI:** 10.3389/fimmu.2019.00590

**Published:** 2019-04-02

**Authors:** Ziv Kaner, Rotem Engelman, Ronen Schuster, Peleg Rider, David Greenberg, Yossef Av-Gay, Moran Benhar, Eli C. Lewis

**Affiliations:** ^1^Department of Clinical Biochemistry and Pharmacology, Faculty of Health Sciences, Ben-Gurion University of the Negev, Beer-Sheva, Israel; ^2^Department of Biochemistry, Rappaport Faculty of Medicine, Technion-Israel Institute of Technology, Haifa, Israel; ^3^The Pediatric Infectious Disease Unit, Soroka University Medical Center, Beer-Sheva, Israel; ^4^Division of Infectious Diseases, Departments of Medicine and Microbiology and Immunology, Life Sciences Institute, University of British Columbia, Vancouver, BC, Canada

**Keywords:** acute phase response, cell activation, cytokines, infection, inflammation, nitric oxide, protease

## Abstract

**Background:** Human α1-antitrypsin (hAAT) is a circulating anti-inflammatory serine-protease inhibitor that rises during acute phase responses. *in vivo*, hAAT reduces bacterial load, without directly inhibiting bacterial growth. In conditions of excess nitric-oxide (NO), hAAT undergoes S-nitrosylation (S-NO-hAAT) and gains antibacterial capacity. The impact of S-NO-hAAT on immune cells has yet to be explored.

**Aim:** Study the effects of S-NO-hAAT on immune cells during bacterial infection.

**Methods:** Clinical-grade hAAT was S-nitrosylated and then compared to unmodified hAAT, functionally, and structurally. Intracellular bacterial clearance by THP-1 macrophages was assessed using live *Salmonella typhi*. Murine peritoneal macrophages were examined, and signaling pathways were evaluated. S-NO-hAAT was also investigated after blocking free mambranal cysteine residues on cells.

**Results:** S-NO-hAAT (27.5 uM) enhances intracellular bacteria elimination by immunocytes (up to 1-log reduction). S-NO-hAAT causes resting macrophages to exhibit a pro-inflammatory and antibacterial phenotype, including release of inflammatory cytokines and induction of inducible nitric oxide synthase (iNOS) and TLR2. These pro-inflammatory effects are dependent upon cell surface thiols and activation of MAPK pathways.

**Conclusions:** hAAT duality appears to be context-specific, involving S-nitrosylation in a nitric oxide rich environment. Our results suggest that S-nitrosylation facilitates the antibacterial activity of hAAT by promoting its ability to activate innate immune cells. This pro-inflammatory effect may involve transferring of nitric oxide from S-NO-hAAT to a free cysteine residue on cellular targets.

## Introduction

Human α1-antitrypsin (hAAT) is a 52 kDa glycoprotein that is mainly produced by hepatocytes, and circulates at steady-state levels of 1.05–1.64 mg/ml (1). During acute phase responses, such as during infection, circulating levels of hAAT rise more than 4-fold (2). Recent findings establish that hAAT also modifies immune cells toward a tolerogenic profile (3–6), while expediting resolution of inflammatory events (7).

Innate immune cells display unique behaviors when exposed to physiologic concentrations of hAAT. For example, stimulated cultured macrophages release interleukin-10 (IL-10) at the expense of interleukin-1-beta (IL-1β), interleukin-6 (IL-6), and Tumor necrosis factor alpha (TNFα) (8). Accordingly, during conditions of high hAAT levels, sera content displays an abrupt shift toward lower IL-1β, IL-6, TNFα and IL-8, and higher IL-10 levels (5, 8–16).

At infection sites, immune cells like macrophages express inducible nitric oxide synthase (iNOS), followed by production of massive quantities of nitric oxide (NO) (17). Providing that local levels of NO are sufficient, it is toxic to bacteria (18). In addition, NO can act as a signaling molecule by promoting S-nitrosylation (S-NO) of both host and pathogen proteins. Protein S-nitrosylation has gained appreciation as a regulator of gene transcription and protein function, as well as of inflammatory and cell survival pathways (19); accordingly, dysregulation of S-nitrosylation is related to a plethora of pathologies (20, 21).

hAAT has a single cysteine residue (position 232) and it was previously shown to undergo S-nitrosylation *in vitro* and *ex vivo* (22). Unlike unmodified hAAT, S-NO-hAAT gains the ability to directly eliminate bacteria (22, 23), while its effect on immune cells has yet to be explored. Here, we generated S-NO-hAAT and explored its effects on immunocyte activities in the context of bacterial infections. Our findings reveal a novel crosstalk between S-NO-hAAT and immune cells, which may alter the immune response to infection at the level of cell signaling and inflammatory output.

## Materials and Methods

### hAAT Nitrosylation and Measurements

hAAT (20 mg/ml or 450 μM; Glassia^TM^, Kamada Ltd., Israel) was reduced by 10 min incubation with 50 mM DTT (Sigma-Aldrich, Israel) at 37°C. Excess DTT was removed using Sephadex G-25 columns (GE Healthcare, Israel) equilibrated with nitrosylation buffer (25 mM HEPES pH 7.4 as a buffer, 0.1 mM EDTA, 0.2 mM diethylenetriaminepentaacetate, 10 μM neocuproine, all three as chelating agents and 100 mM NaCl, all from Sigma-Aldrich). Reduced hAAT was then incubated for 30 min with the NO donor, 1,000 μM diethylamine NONOate (Cayman Chemical, USA) followed by adding additional 500 μM diethylamine NONOate at 37°C for 30 min. After excess NONOate was removed by Sephadex G-25 columns, S-nitrosylation efficiency was calculated by measuring protein concentration using Bicinchoninic acid (BCA) protein assay kit (Santa Cruz Biotechnology, USA) and S-NO content by Saville-Griess assay, as previously described (24). Nitrosylation efficiencies (S-NO/protein ratio) were 63–68%. After production, S-NO-hAAT was aliquoted into dark tubes and stored at −80°C. In all experiments, S-NO-hAAT was compared to untreated hAAT and to GSNO (S-Nitrosoglutathione) as a distinct NO carrier. S-NO-hAAT transnitrosylation measurement was conducted after treating peritoneal macrophages with 100 mM N-ethylmaleimide (NEM, Sigma-Aldrich) for 15 min at room temperature. Then, cells were incubated with S-NO-hAAT. Supernatant samples were collected at indicated time points, and S-NO content was determined by Saville-Griess assay.

### Bacterial Killing Assay

S-NO-hAAT—mediated intracellular bacterial killing assay was carried out using the human monocyte cell line, THP-1, as described elsewhere (25). Briefly, cells were maintained in RPMI 1640 containing 5% heat-inactivated FCS, 25 mM HEPES, 2 mM L-glutamine, 1 mM sodium pyruvate and 1% modified Eagle's medium with non-essential amino acids. For macrophage differentiation, the cells were added 40 ng/ml PMA (Sigma-Aldrich) for 24 h. Logarithmic phase *Salmonella typhi* were opsonized using 10% human serum in rotation for 30 min. The effect of S-NO-hAAT on bacterial infection was assessed using either post-treatment or pre-treatment approach. For pre-treatment, the cells were first treated with 27.5 μM of S-NO-hAAT, hAAT, or GSNO for 24 h. The cells were then washed and introduced to opsonized bacteria (MOI 1:10), followed by a 5 min centrifugation at 800 g and 30 min incubation at 37°C. To eliminate extracellular bacteria, the cells were washed 3 times and incubated for 2 h with 100 mg/ml gentamicin, followed by 12 mg/ml gentamicin containing medium for an additional 4 h. Cells were then washed, and lysed with sterile sodium deoxycholate 0.1% (w/v) in PBS. Lysates were plated on blood agar plates for 24 h at 37°C, and CFU was determined manually.

In the post-treatment protocol, cells were first infected by opsonized bacteria (MOI 1:5). After centrifugation and incubation, the remaining extracellular bacteria were removed by washing and incubation with medium containing 100 mg/ml gentamicin. The cells were treated for 2 h with S-NO-hAAT, hAAT, or GSNO, followed by replacement of supernatant with medium containing 12 mg/ml gentamicin; lysis and CFU counting followed.

### Animals

C57BL/6J female mice (10–12 weeks old) were purchased from Harlan (Jerusalem, Israel) and housed at standard conditions. The study was carried out in accordance with recommendations of the “*1994 law for the prevention of cruelty to animals (experiments on animals)*,” and was approved by Ben-Gurion University of the Negev committee for the ethical care and use of animals in experiments, approval #IL-21-05-2013.

### Peritoneal Macrophage Isolation and Activation

With the exception of the intracellular bacterial killing assay, all experiments were performed using primary peritoneal macrophages. Mice were sacrificed 4 days after 3% thioglycolate (2 ml) peritoneal-injection (i.p.). For peritoneal macrophages collection, 8 ml ice-cold PBS were injected i.p., then recovered using an 18g needle. The cells were counted and left to adhere for at least 2 h in RPMI 1640 supplemented with 10% FCS, 1% L-glutamine, and 1% penicillin-streptomycin (all from Biological Industries, Ltd). *In vitro* activation experiments were carried out in RPMI 1640 supplemented with 5% FCS medium. Cells were treated with 27.5 μM of hAAT, S-NO-hAAT, or GSNO 1 h prior to LPS activation (10 ng/ml, Sigma-Aldrich). At indicated time points, supernatants were collected for analysis and cells were lysed for RNA or protein analysis.

### Cytokine Analysis

Supernatant levels of TNFα, IL-1β, and CXCL-1 were determined by Q-Plex mouse cytokine chemiluminescence-based ELISA (Quansys Biosciences, Logan, UT), according to manufacturer recommendations.

### Real-Time PCR Assays

Total RNA was extracted from cells using total RNA purification kit (Norgen Biotek Corp., Canada), and quantified using NanoDrop spectrophotometer (ND-1000, NanoDrop Technologies, USA). Reverse transcription was performed with the qScriptTM cDNA synthesis kit (Quanta BioSciences, Gaithersburg, MD). Quantification of gene transcription was performed using Fast SYBR Green Master Mix and StepOnePlus Real-Time PCR system (Applied Biosystems, USA). Transcript levels were normalized to GAPDH transcript output.

Primer sequences are as follows (′5 to ′3, FW | RE): **GAPDH**, TCAACAGCAACTCCCACTCTTCCA | ACCCTGTTGCTGTAGCCGTATTCA; **TNFα**, GACCCTCACACTCAGATCATCTTC | CGCTGGCTCAGCCACTCC; **IL-1β**, AAAGCCTCGTGCTGTCGGACC | TTGAGGCCCAAGGCCACAGGTA; **CXCL-1**, AGACCATGGCTGGGATTCAC | AGTGTGGCTATGACTTCGGT; **IL-6**, CCAGTTGCCTTCTTGGGACT | GGTCTGTTGGGAGTGGTATCC; **iNOS**, TTCACTCCACGGAGTAGCCT | CCAACGTTCTCCGTTCTCTTG; **TLR2**, GCATCCGAATTGCATCACCG | CCTCTGAGATTTGACGCTTTGTC.

All primer melting curves had a single clear peak across samples, indicating that their RT-PCR products are single entities (Supplementary Figure 1).

### Kinase Array and Western Blot Analysis

After activation, cells were lysed and phospho-protein levels were compared using human phospho-kinase antibody array (R&D Systems), according to manufacturer guidelines. Array dots were digitally analyzed using the open-source software ImageJ version 1.49 (NIH, USA). Lysate proteins were also denatured and separated on SDS-PAGE, and transferred to PVDF membrane. Western blotting was performed using primary rabbit antibodies anti-Pp38 (sc-17852-R, Santa Cruz Biotechnology), anti-p38 (#9212), anti-PSAPK/JNK (#9251), anti-SAPK/JNK (#9252), anti-PErk1/2 (#9101), anti-Erk1/2 (#9102), all from Cell Signaling Technology (USA) and mouse anti-actin (MAB1501, Merk Millipore, Germany). To detect primary antibody binding, blots were incubated with horseradish-peroxidase-conjugated anti-rabbit or anti-mouse antibodies. The immobilized antibodies were detected by ECL reagent (Advansta, USA).

### Cysteine Blocking and Inhibitors of Signaling Pathways

The involvement of reduced membrane thiols in the activities of S-NO-hAAT was assessed by treating cells with 3 mM 5,5′-dithiobis (2-nitrobenzoic acid) (DTNB) for 30 min at 37°C. p38, JNK and ERK inhibition was executed by pre-treatment for 30 min with 30 μM SB203580, SP600125 and PD98059, respectively (all from Sigma-Aldrich). After either cysteine blocking or signaling inhibition, cells were washed and treated with S-NO-hAAT for 1 h, followed by RNA isolation.

### Statistical Analysis

Analyses were performed using GraphPad Prism 5 software (GraphPad Prism 5, Pugh computers, UK). Results are expressed as the mean ± standard deviation. Significance of differences between groups was determined by two-tail non-parametric Mann-Whitney test. Results are considered significant at *p* ≤ 0.05.

## Results

### S-NO-hAAT Improves Killing of Intracellular Bacteria by Macrophages

**Figure 1:**
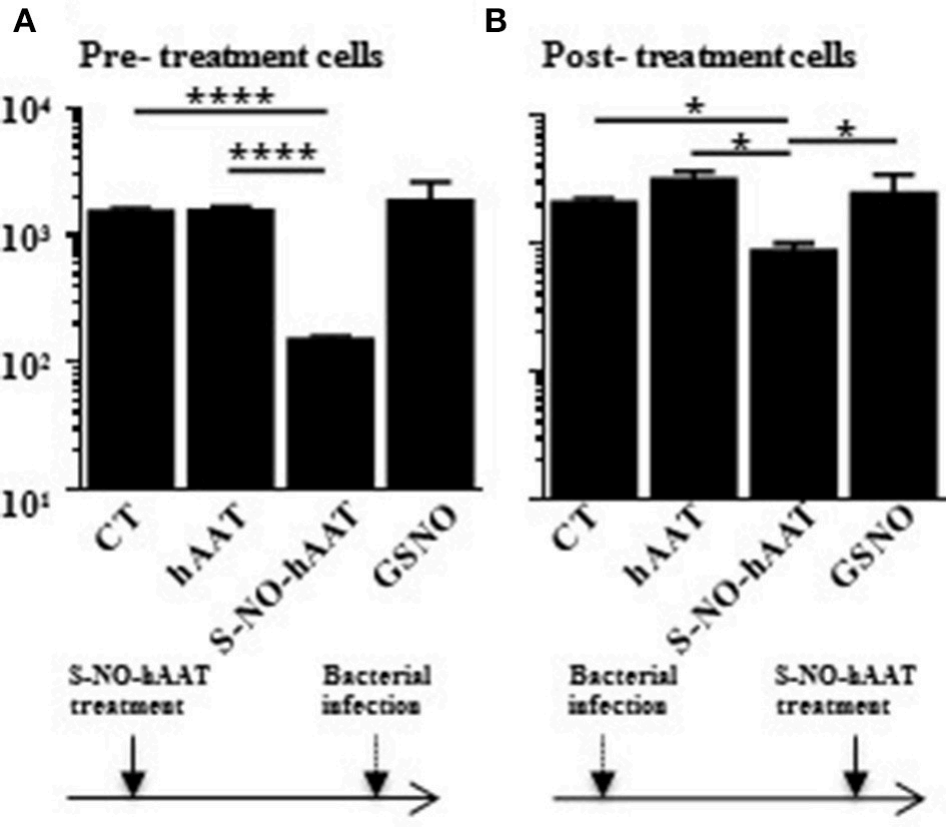
S-NO-hAAT–activated macrophages kill intracellular *Salmonella typhi*. THP-1 cells (0.5 × 10^6^ per well) were pre- **(A)** or post- **(B)** treated with equimolar (27.5 μM) S-NO-hAAT, hAAT, and GSNO. The cells were also infected with live *Salmonella typhi* either prior or after treatment. CT, cells without treatment. In order to eliminate extracellular bacteria, the cells were washed and incubated with gentamicin, as detailed in the methods section. Remaining live bacteria were determined in cell lysates by counting CFU on blood agar, and exhibited logarithmic scale. **(A,B)** are representative results of two independent experiments (*n* = 3) for every condition. Mean ± SD, ^*^p < 0.05 and ^****^p < 0.0001.

S-NO-hAAT was recently found to directly reduce bacterial count. In order to examine the involvement of immunocytes in this phenomenon, macrophage-mediated bacterial killing assay was carried out. Macrophage bacterial killing activity was examined using PMA-primed THP-1 cells infected with *Salmonella typhi* after or prior to treatment with hAAT, S-NO-hAAT, or GSNO (Figure 1). In both settings, analysis of CFU counts obtained from lysed macrophages showed that antibacterial activity was enhanced by S-NO-hAAT. In contrast, non-nitrosylated hAAT and GSNO had no significant effect.

### S-NO-hAAT Increases Macrophage Pro-inflammatory and Anti-bacterial Phenotype

**Figure 2:**
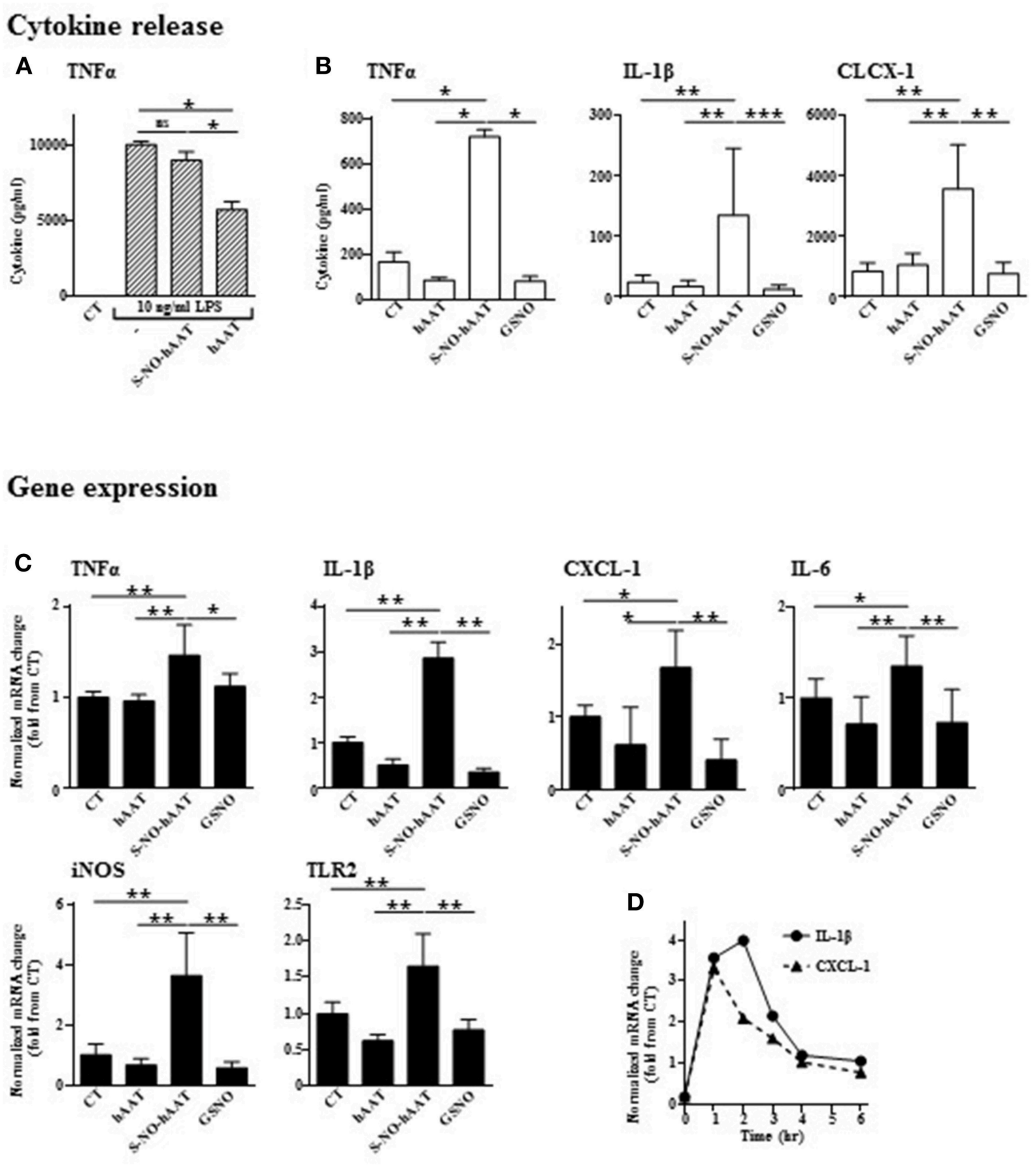
Unlike unmodified hAAT, S-NO-hAAT increases inflammatory responses. Cytokine levels were determined in the supernatants of peritoneal macrophages (0.5 × 10^6^ per well) 48 h post-treatment with equimolar (27.5 μM) S-NO-hAAT, hAAT, GSNO and nitrosylation buffer (CT). **(A)** 10 ng/ml LPS stimulation 1 h post-treatment (*n* = 4). **(B)** S-NO-hAAT, hAAT, and GSNO treatment without added stimulus (*n* = 4). **(C)** mRNA relative levels in peritoneal macrophages (0.25 × 10^6^ per well, *n* = 6 from two independent experiments) 6 h post-treatment with equimolar (27.5 μM) S-NO-hAAT, hAAT, GSNO and nitrosylation buffer (CT). **(D)** Relative mRNA levels post S-NO-hAAT treatment at indicated time intervals. All data are presented as mean ± SD, ^*^p < 0.05, ^**^p < 0.01, and ^***^p < 0.001.

In order to determine whether S-NO-hAAT maintains the anti-inflammatory profile of non-nitrosylated–hAAT, LPS-stimulated peritoneal macrophages were cultured in the presence of S-NO-hAAT for 48 h (Figure 2A). As expected, non-nitrosylated—hAAT significantly inhibited LPS-induced release of TNFα. In contrast, S-NO-hAAT failed to reduce macrophage release of TNFα.

Without added stimulation, macrophages were exposed for 48 h to either S-NO-hAAT, hAAT, or GSNO (all at 27.5 μM), and thereafter inflammatory mediators were measured. As shown, unlike hAAT or GSNO, S-NO-hAAT significantly increased the released levels of TNFα, IL-1β, and CXCL-1 (Figure 2B) as well as inflammatory genes expression after 6 h (Figure 2C). The kinetics of this phenomenon on IL-1β and CXCL-1 gene expression, as representative pro-inflammatory genes, appears to be rapid and short-lived (Figure 2D).

### S-NO-hAAT Activates MAPK Pathways in Macrophages

**Figure 3:**
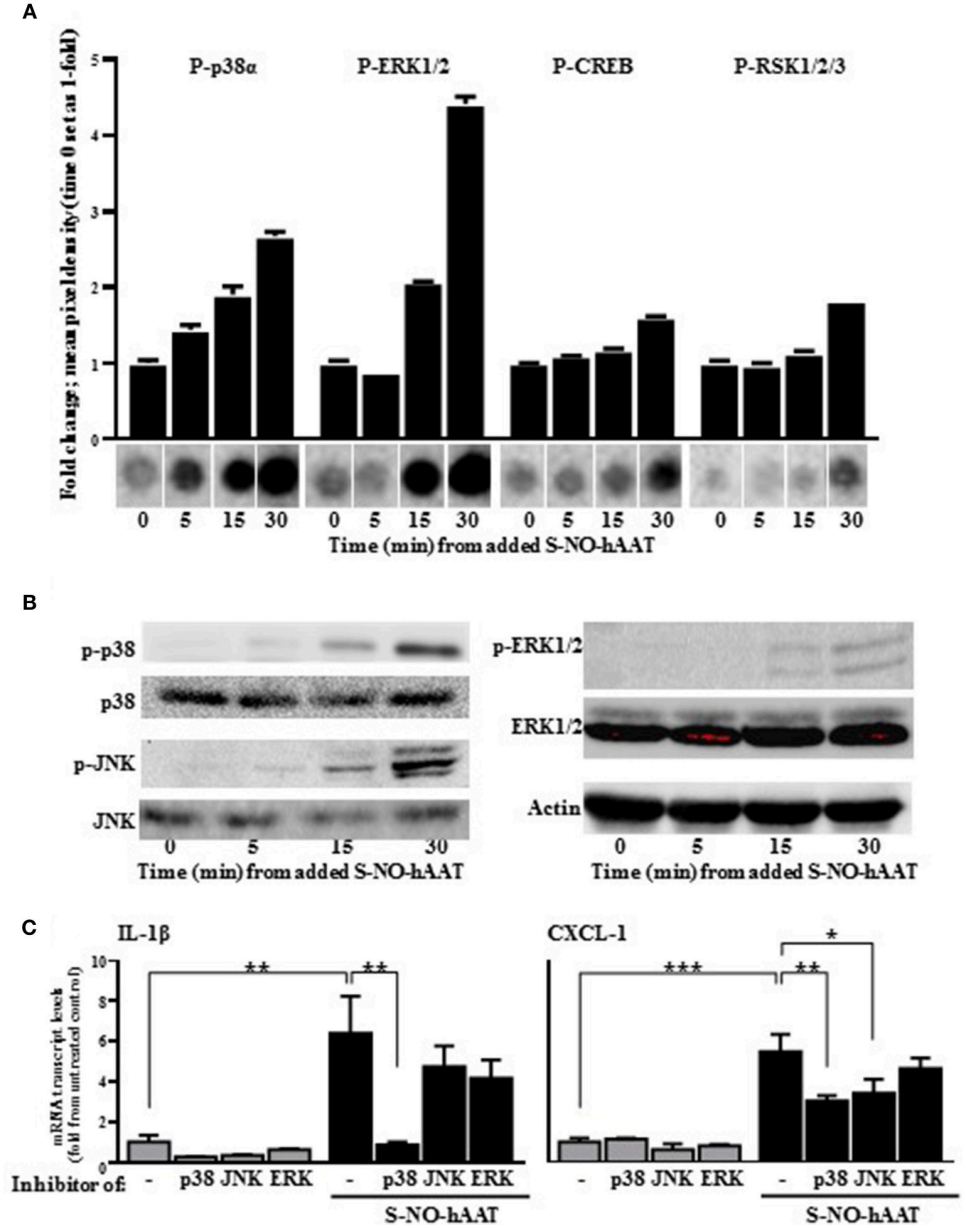
S-NO-hAAT activates MAPK signaling pathways. Peritoneal macrophages lysate (1 × 10^7^ per well) after incubation with 27.5 μM S-NO-hAAT for indicated time periods. **(A)** Kinase array, performed once for each time interval. Graph, densitometry analysis, mean ± SD. Below, representative assay blots. **(B)** Representative Western blot analysis of MAPK signaling proteins. **(C)** mRNA transcript levels of IL-1β and CXCL-1 of naïve (gray) or 1 h S-NO-hAAT treated (black) peritoneal macrophages (0.25 × 10^6^ per well) in the presence of signaling inhibitors or DMSO (–). mRNA transcript levels normalized to GAPDH (*n* = 3). Data are presented as mean ± SD. ns, non-significant, ^*^p < 0.05, ^**^p < 0.01, and ^***^p < 0.001.

In order to validate the authenticity of S-NO-hAAT pro-inflammatory effect, signaling pathways activation were analyzed on peritoneal macrophages. Phosphorylation of proteins in the MAPK cascade was evaluated, using kinase array and western blot analysis of the cell lysates. S-NO-hAAT-treated macrophages displayed a rapid (5 min) rise in p38 and JNK phosphorylation. JNK phosphorylation was observed only by Western blot. ERK phosphorylation was observed after 15 min and the transcription factors CREB and RSK appeared to be phosphorylated after 30 min (Figures 3A,B). Phosphorylation was not observed after S-NO-hAAT treatment in the other arrayed proteins (Akt, AMPK, Catenin, Chk-2, c-jun, EGF R, eNOS, FAK, Fgr, Fyn, GSK-3, Hck, HSP27, HSP60, Lck, Lyn, MSK1/2, p27, p53, p70 S6 Kinase, PDGF, PLCγ-1, PRAS40, Pyk2, Src, STAT2/3/5/6, TOR, WNK-1, Yes).

In order to further validate these data, pretreatment of the peritoneal macrophages with MAPK inhibitors was performed prior to S-NO-hAAT introduction. With regards to inflammatory gene expression, both IL-1β and CXCL-1 transcript levels increased several-fold in the presence of S-NO-hAAT (Figure 3C). However, this inducible profile was significantly diminished in the presence of a p38 inhibitor. In addition, treatment with a JNK inhibitor inhibited S-NO-hAAT—mediated induction of CXCL-1, although the induction of IL-1β transcript levels did not change in a statistically significant manner.

### S-NO-hAAT Acts on Cells by Transnitrosylation

**Figure 4:**
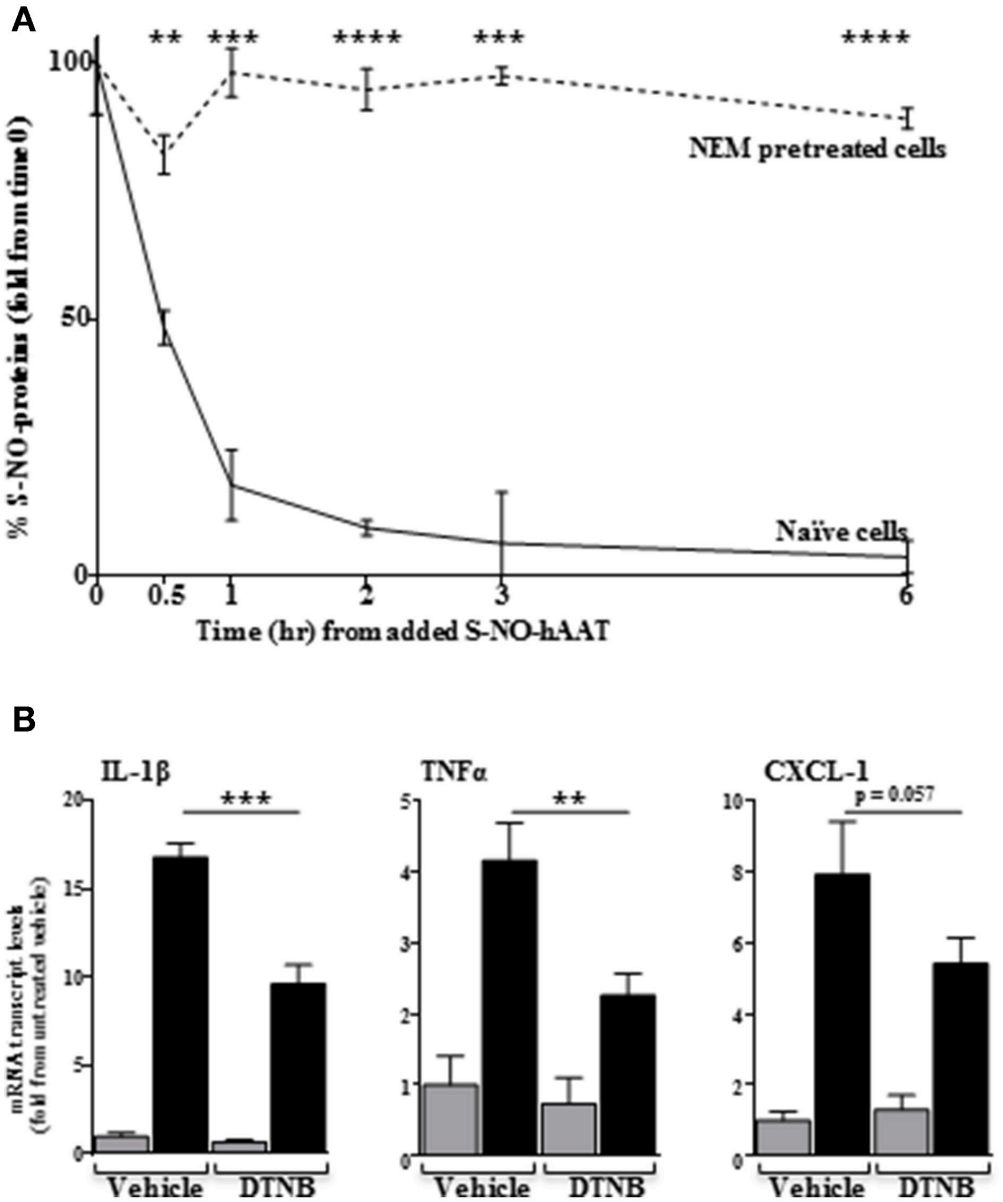
Transnitrosylation-dependent activity of S-NO-hAAT. **(A)** Amount of remaining nitrosylated proteins in supernatant following S-NO-hAAT introduction to naïve (solid) and NEM-pretreated (dashed) peritoneal macrophages (1 × 10^6^ per well, *n* = 3). **(B)** DTNB or DMSO (vehicle)-pretreated peritoneal macrophages (0.25 × 10^6^ per well, *n* = 3) were examined for mRNA levels without S-NO-hAAT (gray) or 1 h after introducing it (black). Data are presented as mean ± SD, ^**^p < 0.01, ^***^p < 0.001, and ^****^p < 0.0001.

We considered the possibility that some activities of S-NO-hAAT may involve transnitrosylation, namely, the transfer of NO molecules from S-NO-hAAT to cellular targets. In order to investigate this possibility, peritoneal macrophages were treated with NEM, a blocker of free cysteines in cellular proteins. Subsequently, cells were treated with S-NO-hAAT followed by analysis of S-NO content in cell supernatants (mostly S-NO-hAAT). As shown in Figure 4A, the level of nitrosylated proteins in cell supernatants had rapidly declined in the presence of naïve cells, but remained mostly unchanged in cultures pretreated with NEM. These data may indicate the possibility that NO groups are transferred from S-NO-hAAT to cellular targets.

Unlike NEM, DTNB does not enter cells; by using DTNB pre-conditioned macrophages, we explored the possibility that S-NO-hAAT transnitrosylates surface targets. As shown in Figure 4B, the inflammatory response elicited by S-NO-hAAT was significantly compromised by DTNB, suggesting that S-NO-hAAT activities depend upon availability of free thiols on cell surfaces.

## Discussion

Macrophages are part of the innate immune system, recognizing, engulfing and destroying potential pathogens, including bacteria. As our results indicate, S-NO-hAAT reduces intracellular bacterial load. The question whether this effect is caused directly by NO molecules carried by S-NO-hAAT, or is an indirect effect facilitated by antibacterial activity of immunocytes, is raised. The observed difference between S-NO-hAAT and the same amount of NO molecules, bound to cysteine on a different carrier (GSNO), suggests that bacterial burden reduction was indirect, possibly a result of altered macrophage activity. Another supporting evidence is that macrophages that were pretreated with S-NO-hAAT (followed by washing) prior to bacterial introduction successfully reduced the bacterial load (Figure 1A). Additionally, unlike in some other reports (22, 23), our results suggest that a direct antibacterial effect is achieved using supra-physiological concentrations of S-NO-hAAT (> 20 μM, not shown). This difference in the direct anti-bacterial activity may be the result of disparity in bacterial strains across studies, as well as the source of hAAT and hAAT S-nitrosylation procedure parameters.

hAAT augmentation therapy is afforded to patients with genetic hAAT deficiency (26), and improves long-term clinical outcomes (27). While *in vivo* experiments are indeed warranted with regards to the prospect of S-NO-hAAT as a therapeutic, our results agree with antibacterial effects of hAAT augmentation therapy observed in several independent clinical studies (28, 29), and to high phagocytosis rate in hAAT-treated alveolar macrophages (30). These studies counter the concern that hAAT supplementation will increase the risk for opportunistic infections, although the mechanism for this is yet unknown.

How may an anti-inflammatory protein increase bacterial clearance via macrophages? Unlike unmodified hAAT or GSNO, our study shows that S-NO-hAAT facilitates the inflammatory state of macrophages by upregulating pro-inflammatory molecules. Although a similar pattern was detected in the levels of pro-inflammatory genes expression and proteins release, it should be noted that the amount of cytokines in the supernatant needs to be examined after shorter time intervals following S-NO-hAAT's introduction, in future studies. The enhancement of macrophages antibacterial activity may be explained by S-NO-hAAT—dependent induction of host-response genes, such as iNOS and TLRs (31). THP-1 cells were previously shown to express iNOS upon exposure to heat-killed Methicillin-resistant strains of *Staphylococcus aureus* (MRSA) (32), granulocyte macrophage colony-stimulating factor (GM-CSF) (33), LPS and silica (34). Future studies would need to address the specific and detailed mechanism of bacterial eradication in various cell lines.

hAAT seems to exert complex effects on the specifics of an inflammatory event, by either promoting or suppressing cellular inflammatory responses. For example, hAAT has a highly consistent inhibitory profile with respect to soluble levels of TNFα (11, 35). However, as our results indicate, S-NO-hAAT can also *promote* the release of TNFα. Some other studies support this duality: we recently reported that following bacterial infection, inflammation, and neutrophil infiltration are increased in hAAT-expressing mice shortly after infection, resulting in significantly reduced bacterial burden *in vivo*. At later time points, immune cell activation and pro-inflammatory mediators were alleviated and overall tissue and organ damage was minimized (9). Additional *in vitro* and *in vivo* evidences to the duality of hAAT may be found in studies that display a short pro-inflammatory burst after hAAT and LPS treatment, such that was *higher* than after LPS treatment alone, followed by an anti-inflammatory wave (36, 37).

While hAAT can exert anti-apoptotic effects (13, 38–40), outcomes of S-NO-hAAT on cell survival are not yet elucidated. Our results indicate that despite the pro-inflammatory phenotype that follows S-NO-hAAT introduction, macrophage survival remained unaffected (Supplementary Figure 2). *In vivo*, S-NO-hAAT has a cytoprotective effect in the context of ischemia-reperfusion injury (41). In contrast, murine lymphoma cell line (RMA) readily expire upon exposure to S-NO-hAAT (31). Therefore, it seems that the effect of S-NO-hAAT on cell survival depends on cell type and surrounding conditions; further studies are needed.

The site of S-nitosylation on hAAT is highly conserved—a single cysteine residue at position 232 (Supplementary Table 1). This cysteine is surrounded by three positively-charged lysines (positions 233, 234 and 274) (42) that may promote deprotonation of its thiol (43). Although S-nitrosylation on the cysteine residue in hAAT was not yet observed *in vivo*, it is likely to occur considering its extremely low pKa (44) and reactivity (42). Interestingly, a mutation of cysteine 232 to proline, results in a superior anti-inflammatory profile of hAAT that lacks serine-protease inhibition activity (45). In contrast, S-nitrosylation of cysteine 232 does not interfere with its inhibition capacity of serine-proteases, found to be similar to (23). Nonetheless, it appears that S-NO-hAAT gains activities that non-nitrosylated hAAT lacks, such as inhibiting several cysteine-proteases (23). Although S-nitrosylation of hAAT does alter its function, the protein remains a 52 kDa protein; that said, it may undergo a structural change (Supplementary Figure 3), an outcome observed in other nitrosylated circulating proteins, including albumin (46), and hemoglobin (47). However, our structural assessment should be elaborated by more structural methods.

MAPK pathways are important for many cell functions, including inflammation, and seem to play a major role in the activity of S-NO-hAAT. Our results indicate that S-NO-hAAT affects MAPK members, especially by activation of p38 and JNK; both phosphorylated within 5 min following S-NO-hAAT treatment, and their inhibition, especially p38, appeared to have significantly reduced S-NO-hAAT-mediated induction of pro-inflammatory genes. These correlations come in contrast with the inhibitory effect of hAAT on p38 (39, 48), as well as inflammation modulation after S-nitrosylation of macrophage proteins (19). In this regard, S-nitrosylation was previously reported to inhibit apoptosis signal-regulating kinase 1 (ASK1),which acts upstream to JNK and p38 (49). On the other hand, S-nitrosylation of neuronal p38 alters its activity according to the source of NO (50). As such, it is unknown whether p38 or JNK are S-nitrosylated by S-NO-hAAT, and it remains to be elucidated exactly how S-NO-hAAT activates p38 and JNK.

What might be the molecular targets of S-NO-hAAT? One possible target may be the scavenger receptor, LDL receptor-related protein 1 (LRP1, also known as CD91), a receptor for complexed hAAT (51, 52) that binds and internalizes hAAT (53). LRP1 also binds LDL and DAMPs, such as gp96 (51, 54, 55). Surfactant D (SP-D), an extracellular protein that is abundant in the lungs and turns inflammatory instead of anti-inflammatory upon S-nitrosylation (56–59), is modified through S-nitrosylation (S-NO-SP-D) and increases inflammation (60–62). After being S-nitrosylated on two cysteine residues, S-NO-SP-D complex disintegrates into trimeric subunits, that then bind and activate LRP1 (58, 63, 64). As a result, LRP1 activates p38-dependent pro-inflammatory signaling pathways. Therefore, LRP1 is a possible target of S-NO-hAAT, although more studies are needed to clarify this prospect.

The kinetics of S-NO-hAAT-induced cytokine expression are not obvious. S-NO-hAAT induced maximal cytokine expression within 1–2 h, which had then sharply declined. We speculate that this short-lived effect may be due to rapid reduction in S-NO-hAAT levels with concomitant emergence of transnitrosylated targets. Indeed, our results indicate that S-NO-hAAT is rapidly de-nitrosylated upon its interaction with its target cells. Moreover, blocking of free cell surface cysteine residues by DTNB reduced the inflammatory flare triggered by S-NO-hAAT, suggesting that the transnitrosylation process of S-NO-hAAT is essential for its pro-inflammatory effect.

The hypothesis by which S-NO-hAAT targets are present on cell membranes is supported by several other studies; hAAT has been shown to dock onto membrane lipid rafts (65), which usually contain multiple inflammatory receptors. hAAT has several hydrophobic domains and directly interacts with cholesterol (66, 67), and exerts a synergistic cytoprotective effect together with HDL (68, 69). hAAT was shown to readily enter the cytosol (53, 70, 71), but according to the rapid effect of S-NO-hAAT, it is more likely that S-NO-hAAT has membrane-associated targets, that unmodified hAAT might not interact with.

**Figure 5:**
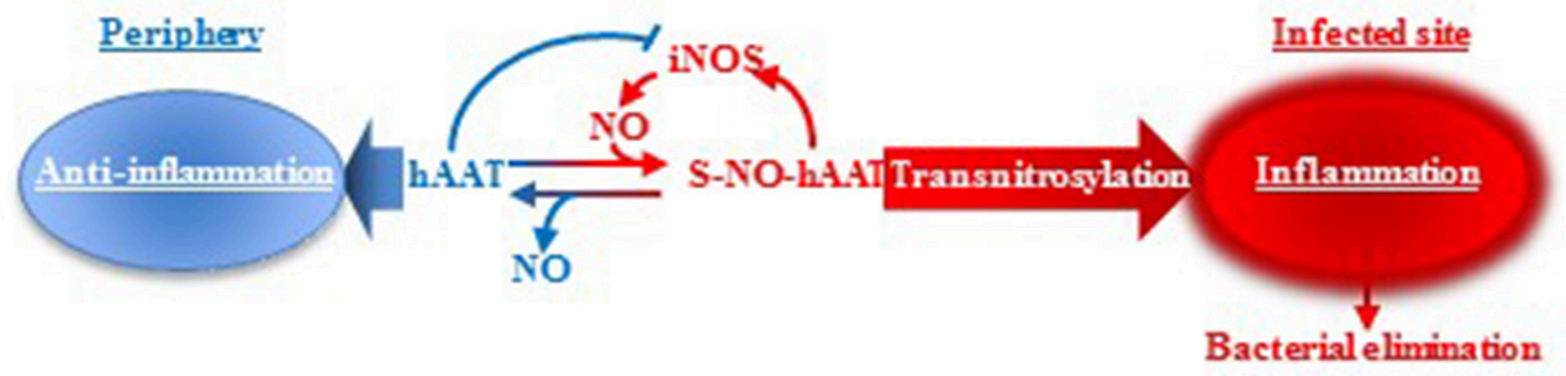
hAAT duality: proposed mechanism. It is suggested that hAAT acts in two different manners according to its S-nitrosylation state. In an infected, inflamed, nitric oxide-rich site, hAAT is nitrosylated and can reduce the bacterial load by acting as an inflammatory trigger for immune cells. However, in the periphery, nitric oxide levels are low and hAAT is presumed to maintain its unmodified anti-inflammatory and tissue-protective activity profile.

Although we do not know the precise proportion of S-NO-hAAT generated *in vivo*, nor the ratio between hAAT/ S-NO-hAAT in the periphery or at infection site, we propose a putative mechanism of action upon S-nitrosylation during bacterial infections (Figure 5). In brief, unlike non-nitrosylated hAAT (13, 72), S-NO-hAAT increases iNOS expression, suggesting there exists a local pro-inflammatory positive feedback loop. hAAT is an abundant protein in the circulation, with distinct context-specific activities. According to this concept, in the periphery, hAAT most probably remains in its unmodified form and acts as an anti-inflammatory and tissue-protective agent. However, during infection, infiltrating and resident immune cells become activated, iNOS expression is induced and local nitric-oxide levels dramatically rise; it is postulated that hAAT that reaches a site of infection is thus S-nitrosylated, and can assist in the reduction of the bacterial burden by further activating immune cells. The rise in iNOS expression has a potential to form more S-NO-hAAT upon entry of unmodified hAAT to the site of infection, in accordance with the prototypical elevation in circulating hAAT levels during infection. Nitric oxide may then be transferred from S-NO-hAAT to immune cell-associated proteins by direct transnitrosylation, thus reducing the probability of residual inflammatory S-NO-hAAT in the periphery.

**Figure 6:**
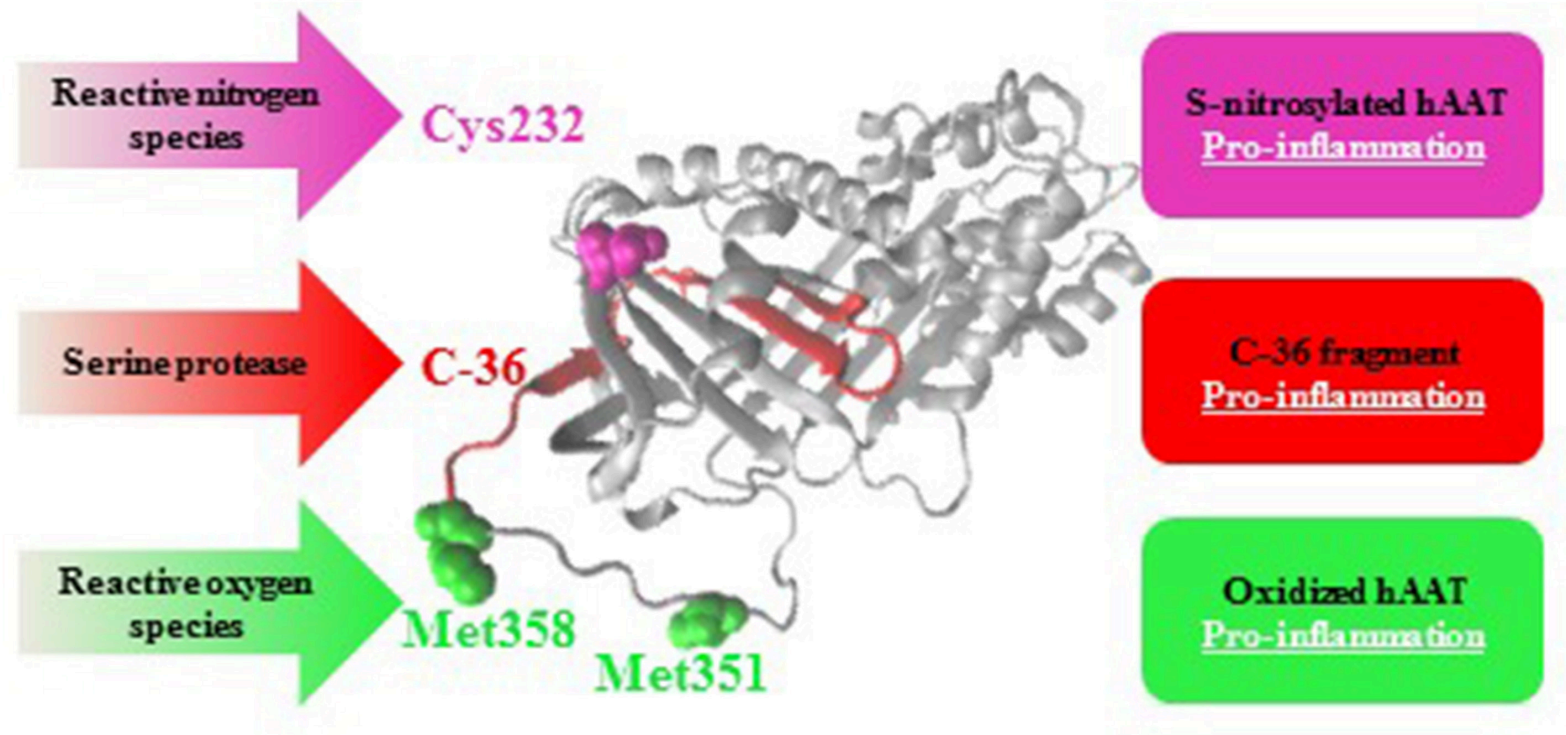
Post-translational modifications in hAAT: pro-inflammatory outcomes. As an acute phase protein that rises during inflammation, hAAT may turn pro-inflammatory in an infected site as a result of S-nitrosylation (Cys232, pink), oxidation (Met351 and Met358, green), or proteolytic cleavage followed by release of its 36 amino-acids carboxyl terminal (C-36, red).

In addition to S-nitrosylation, hAAT is known to undergo two more post-translational modifications at inflamed sites: its proteolytic cleavage, and an oxidation process (Figure 6). hAAT oxidation is a reversible modification mediated by reactive oxygen species that are typically abundant at an inflamed tissue. The oxidation of hAAT occurs on two methionine residues, Met351 and Met358 (73), and oxidized hAAT turns inflammatory toward monocytes (74) and epithelial cells (75). The proteolytic cleavage of hAAT results in the release of a 36 amino-acid long peptide (C-36) from the carboxyl terminus of hAAT; C-36 activates human monocytes (76, 77) and facilitates human neutrophil chemotaxis and degranulation (78). Indeed, S-nitrosylation, oxidation and proteolytic-cleavage occur primarily in inflammatory conditions, such that contain excessive levels of free radicals and proteases, and under circumstances that elevate hAAT levels in the whole organism.

To conclude, S-nitrosylation of hAAT represents a physiological post-translational modification, which alters the function of hAAT from an anti- to a pro-inflammatory protein, through, at least in part, activation of MAPK signaling pathways. S-NO-hAAT is not an antibiotic; unlike antibiotics, indirect facilitation of immunocytes toward pathogen elimination possesses a therapeutic potential with lower chances of bacterial resistance emergence. Further investigation is required in order to fully understand the dynamics and mechanism of action of S-NO-hAAT, and the full spectrum of its possible clinical applications.

## Data Availability

All datasets generated for this study are included in the manuscript and/or the Supplementary Files.

## Ethics Statement

Experiments were approved by the Institutional Animal Care and Use Committee.

## Author Contributions

ZK, RE, RS, and PR performed the experiments described in the study. ZK, RE, DG, YA-G, MB, and EL contributed to the conception and design of the study. ZK and RS performed the statistical analysis. ZK organized the data and wrote the first draft of the manuscript. ZK, RS, PR, YA-G, MB, and EL wrote sections of the manuscript. All authors contributed to revising the manuscript, reading and approving the submitted version.

### Conflict of Interest Statement

The authors declare that the research was conducted in the absence of any commercial or financial relationships that could be construed as a potential conflict of interest.
