# Supplementary material for: S-Nitrosylation of α1-Antitrypsin Triggers Macrophages Toward Inflammatory Phenotype and Enhances Intra-Cellular Bacteria Elimination
Source: Front Immunol. 2019 Apr 2;10:590. doi: 10.3389/fimmu.2019.00590 (PMC6454134; doi:10.3389/fimmu.2019.00590)
Supplement: Supplementary file 1 [file Table_1.pdf]

Supplementary Table 1: Mammalian antitrypsin-like sequences alignment analysis.

Mammalian AAT-like sequences were collected using the NCBI BLASTp web interface with default parameters; query sequence was human AAT variant version ABG73380.1. For database option, a non-redundant protein sequence (nr) was chosen and 19 more random sequences were chosen from diverse mammals. For Multiple Sequence Alignment (MSA), amino acid sequences were inserted in FASTA format, and analyzed by Clustal Omega [1].

The table represents a comparison between hAAT and AAT-like sequences. The single cysteine residue and its 3 proximal lysine residues were compered between the different sequences. Dark gray represents complete identity to hAAT amino acids sequence, pale gray represents similarity according to the amino acid characterization.

Consistent reoccurrence of a semi-surface single cysteine residue at position 232 of hAAT was noticed among all examined mammals. This analysis further shows that the positively charged amino acid, Lys274, located proximally to Cys232 in the tertiary structure, exhibits a 95% similarity. Our in-silico comparison, indicates that although Cys232 does not participate in an intra-protein S-S bond nor within the prototypical reactive center loop (RCL), it is highly conserved.

Organism name	Accession code	Total sequence Identity	Amino acid position			
			**232**	**233**	**234**	**274**
Homo sapiens	ABG73380	100%	**C**	**K**	**K**	**K**
Pan paniscus	XP_003832864.1	99%	C	K	K	K
Macaca mulatta	NP_001252946.1	91%	C	E	K	K
Canis lupus familiaris	NP_001073578.2	75%	C	A	T	K
Sus scrofa	NP_999560.1	74%	C	D	K	K
Physeter catodon	XP_007118979.1	74%	C	D	K	K
Pteropus Alecto	XP_006925619.1	73%	C	D	T	K
Tursiops truncatus	XP_004320745.1	73%	C	D	R	K
Odobenus rosmarus divergens	XP_004394562.1	73%	C	D	K	K
Myotis davidii	XP_006768021.1	72%	C	H	K	M
Felis catus	XP_006933138.1	72%	C	D	M	K
Rattus norvegicus	NP_071964.2	69%	C	S	T	R
Ovis aries	NP_001009799.1	69%	C	D	K	K
Bos Taurus	NP_776307.1	69%	C	D	K	K
Lipotes vexillifer	XP_007447379.1	68%	C	D	R	K
Mus musculus	NP_033270.3	64%	C	S	I	K
Oryctolagus cuniculus	NP_001075666.1	64%	C	S	T	K
Erinaceus europaeus	XP_007529139.1	64%	C	D	T	K
Ceratotherium simum simum	XP_004434291.1	56%	C	D	T	R
Mustela putorius furo	XP_004754804.1	44%	C	N	K	K

1. Li, W., et al., *The EMBL-EBI bioinformatics web and programmatic tools framework.* Nucleic Acids Res, 2015. **43**(W1): p. W580-4.
