# Supplementary figures and images for: S-Nitrosylation of α1-Antitrypsin Triggers Macrophages Toward Inflammatory Phenotype and Enhances Intra-Cellular Bacteria Elimination
Source: Front Immunol. 2019 Apr 2;10:590. doi: 10.3389/fimmu.2019.00590 (PMC6454134; doi:10.3389/fimmu.2019.00590)

Supplementary figure 1: RT-PCR primers melting curves.

GAPDH:

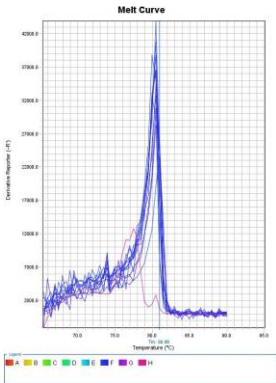

IL-1β:

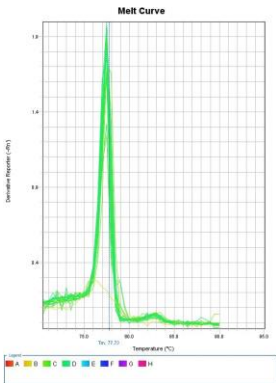

IL-6:

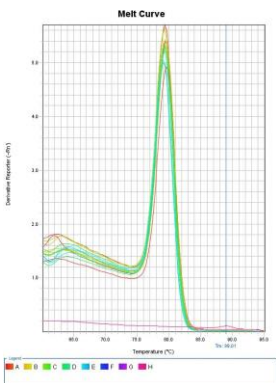

**CXCL-1:**

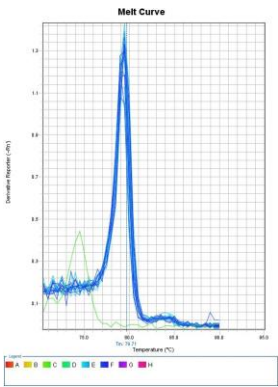

**TNF $\alpha$**

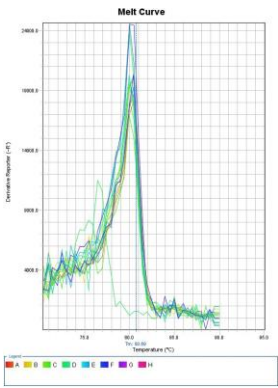

**iNOS:**

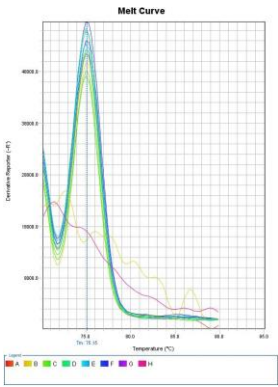

## TLR2:

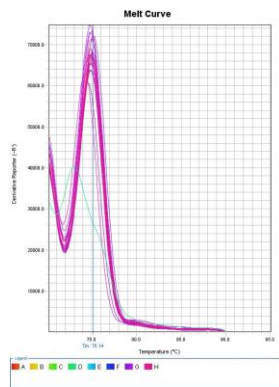

Supplement: Supplementary file 2 [file Image_1.pdf]
