# Supplementary material for: S-Nitrosylation of α1-Antitrypsin Triggers Macrophages Toward Inflammatory Phenotype and Enhances Intra-Cellular Bacteria Elimination
Source: Front Immunol. 2019 Apr 2;10:590. doi: 10.3389/fimmu.2019.00590 (PMC6454134; doi:10.3389/fimmu.2019.00590)
Supplement: Supplementary file 3 [file Image_2.pdf]

Supplementary figure 2: Nitrosylated hAAT does not cause macrophage death.

In order to investigate that S-NO-hAAT does not cause macrophage death, we assessed S-NO-hAAT toxicity towards macrophages. THP-1 cells (0.3×10^6 per well) were incubated for 24-hours with 27.5 µM of hAAT or S-NO-hAAT, or 10 µM Cytarabine (Sigma-Aldrich) as a positive control. Cell viability was assessed by supernatant relative LDH content (cytotox-96 kit, Promega), and AnnexinV-FITC/PI staining (BioLegend). Flow cytometric experiments were done using BD Canto II (BD Biosciences, Singapore) and data were analyzed by FLOWJO 7.6.3 software (Flowjo, LLC Data Analysis Software, Ashland, OR).

As our Cytarabine treatment indicates, Higher levels of supernatant LDH or double positive AnnexinV-FITC/PI cells (both are presented as mean ± SEM) are correlated with more dead cells. In contrast with the cytarabine treatment, the amount of surviving cells was high and comparable between S-NO-hAAT, hAAT and untreated cells, indicating that S-NO-hAAT does not cause macrophage death.

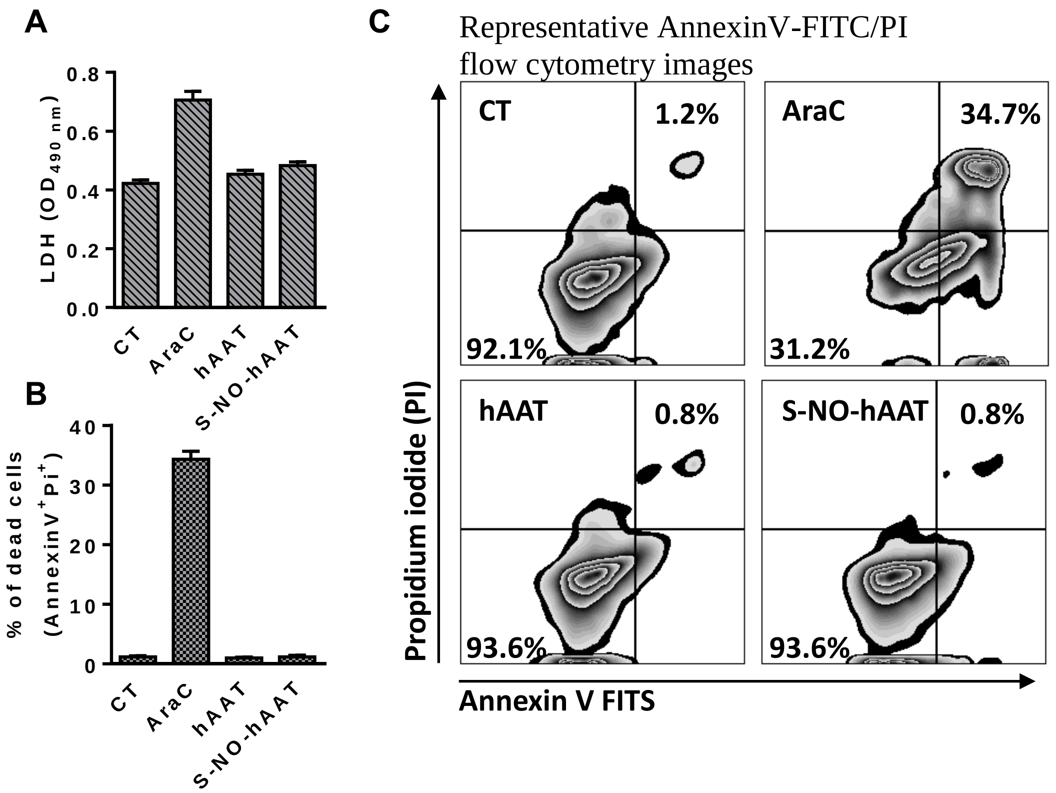

A
0.8
0.6
0.4
0.2
0.0
LDH (OD490 nm)
CT
AraC
hAAT
S-NO-hAAT
B
40
30
20
10
0
% of dead cells (AnnexinV+Pi+)
CT
AraC
hAAT
S-NO-hAAT
C
Representative AnnexinV-FITC/PI flow cytometry images
CT
1.2%
92.1%
AraC
34.7%
31.2%
hAAT
0.8%
93.6%
S-NO-hAAT
0.8%
93.6%
Propidium iodide (PI)
Annexin V FITS
