# Supplementary material for: S-Nitrosylation of α1-Antitrypsin Triggers Macrophages Toward Inflammatory Phenotype and Enhances Intra-Cellular Bacteria Elimination
Source: Front Immunol. 2019 Apr 2;10:590. doi: 10.3389/fimmu.2019.00590 (PMC6454134; doi:10.3389/fimmu.2019.00590)

Supplementary figure 3: Biochemical properties of S-NO-hAAT are distinct from non-nitrosylated hAAT.

In order to further characterize S-NO-hAAT in comparison to hAAT, their physical attributes were analyzed. 20 mg of unmodified and nitrosylated hAAT were analyzed on 12% native and SDS denaturative acrylamide gel, followed by Coomassie brilliant blue staining. For native PAGE, the proteins were first diluted with sample buffer (0.2 M Tris-HCl pH 6.8, 10% v/v Glycerol and 0.2% Bromophenol blue) and separated on ice using cold running buffer, without supplement of SDS or reducing reagents. The SDS-PAGE analysis was carried out after 5 minutes of boiling (95°C) the samples with Laemmli sample buffer.

Differential scanning calorimetry (DSC) experiments were performed on a VP-DSC (MicroCal, NJ, USA). hAAT and S-NO-hAAT were diluted to a final concentration of 0.5 mg/ml and a volume of 0.6 ml. Nitrosylation buffer served as a blank. Scan rates of 1°C/ minute and high gain were employed. Each sample was scanned three times. Data analysis was performed using Microcal Origin 7.0 software.

As shown in **Supplementary figure 2A**, S-NO-hAAT migrated in a manner consistent with reduced hAAT under denaturing conditions (SDS-PAGE); however, in native gel analysis, S-NO-hAAT migrated in a slightly distinct manner. This observation suggests that S-nitrosylation may cause hAAT to undergo a structural change. We assessed protein stability using differential scanning calorimetry (DSC) and found that S-NO-hAAT has reduced stability compared to the non-nitrosylated proteins (**Supplementary figure 2B, left**). For both hAAT and S-NO-hAAT thermal-denaturation-plots are relatively asymmetric and displayed two denaturation peaks, likely reflecting partial and complete unfolding of the proteins (**Supplementary**

figure 2B, right). However, those are preliminary results, and more studies are needed (such as crystallography) to analyze the differences between S-NO-hAAT and unmodified hAAT structures.

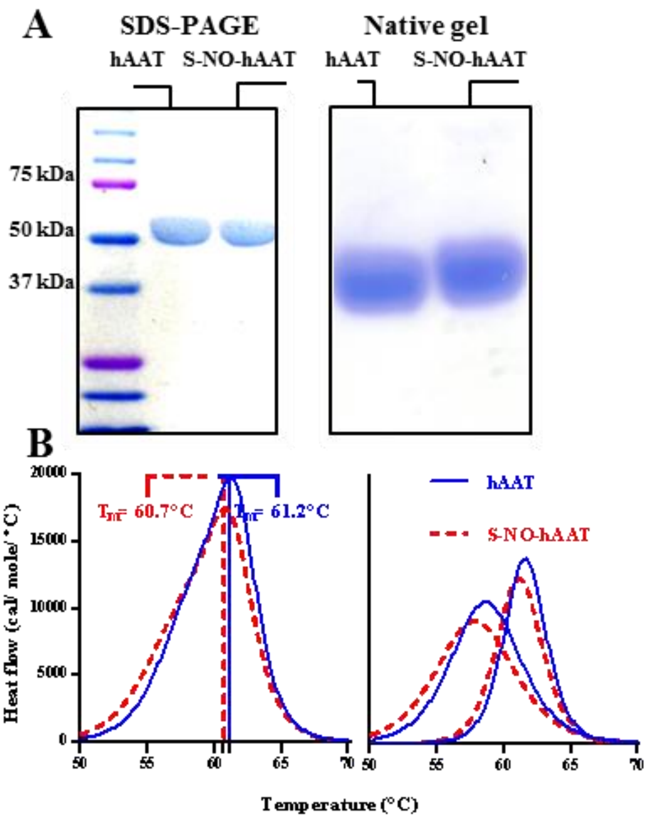

Supplement: Supplementary file 4 [file Image_3.pdf]
